# Supplementary figures and images for: A genome-wide association study (GWAS) of the personality constructs in CPAI-2 in Taiwanese Hakka populations
Source: PLoS One. 2023 Feb 17;18(2):e0281903. doi: 10.1371/journal.pone.0281903 (PMC9937499; doi:10.1371/journal.pone.0281903)

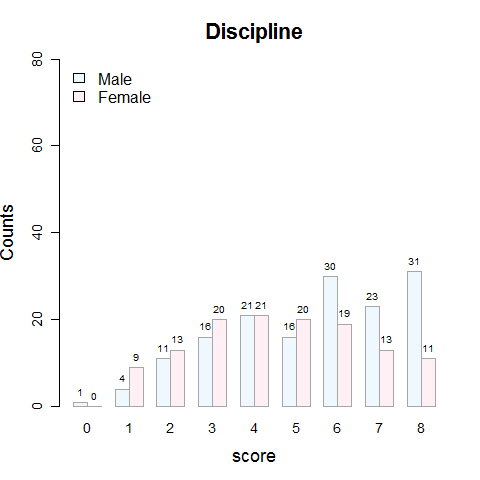

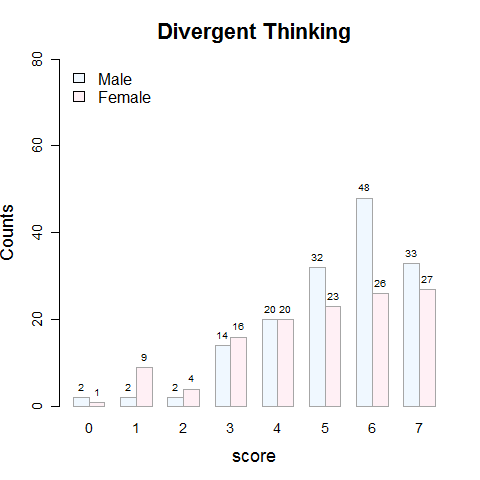

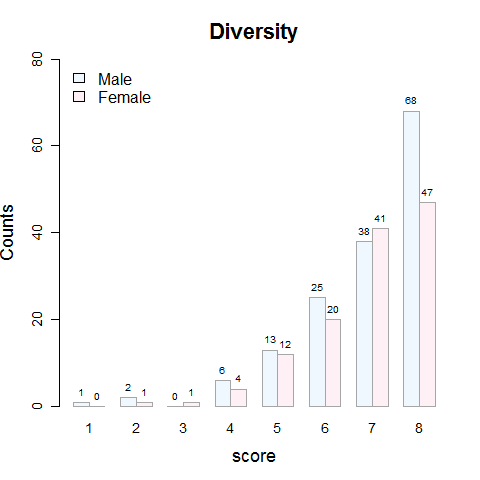

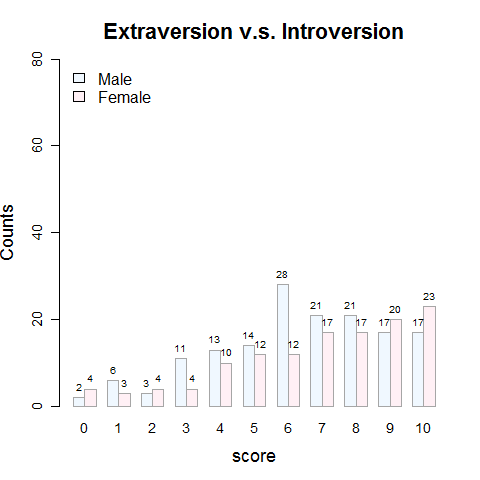

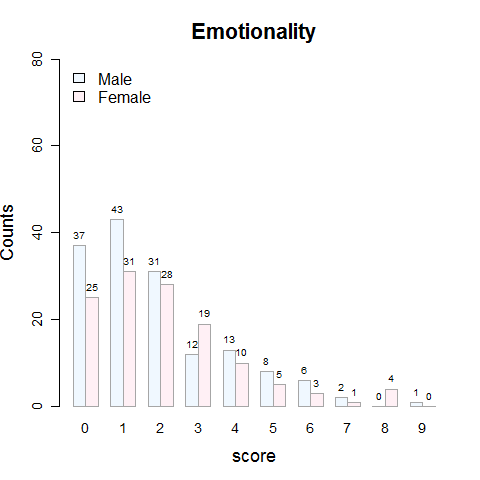

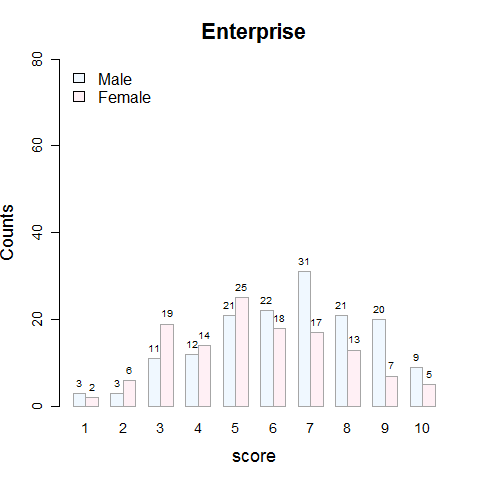

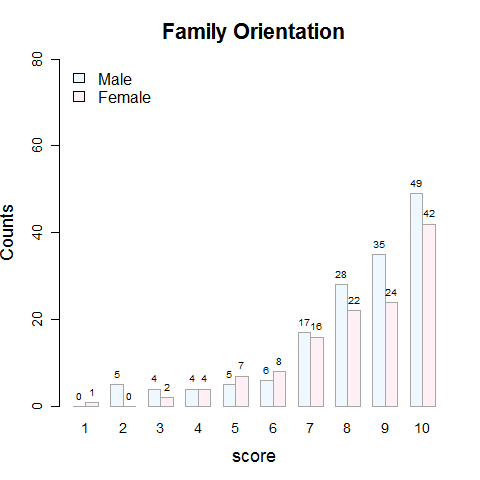

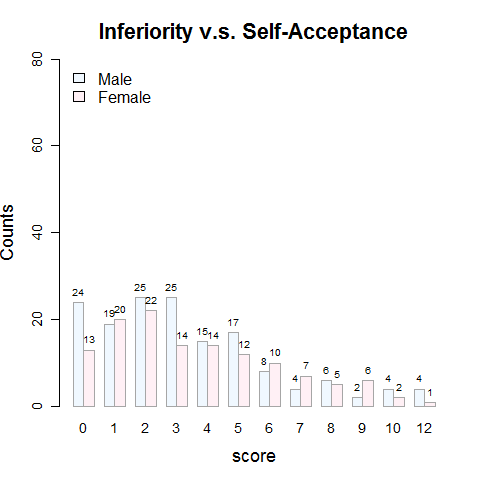

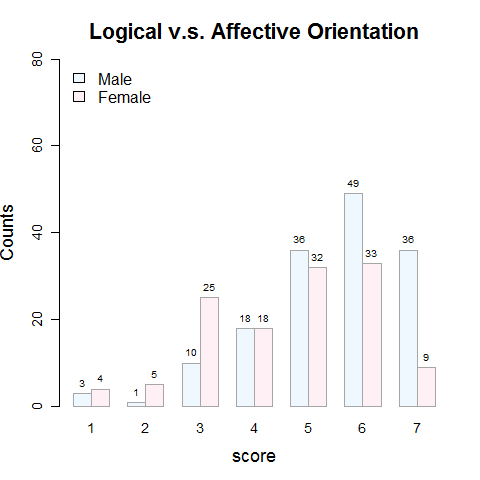

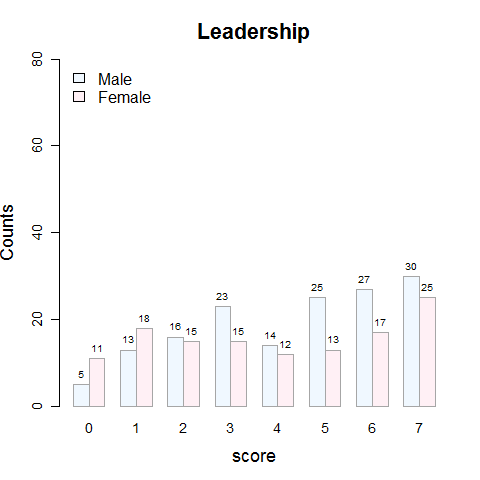

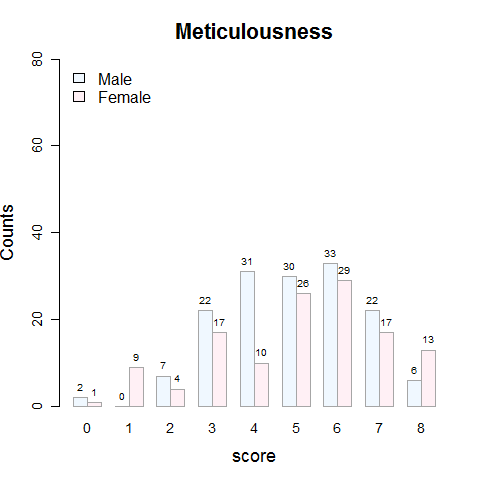

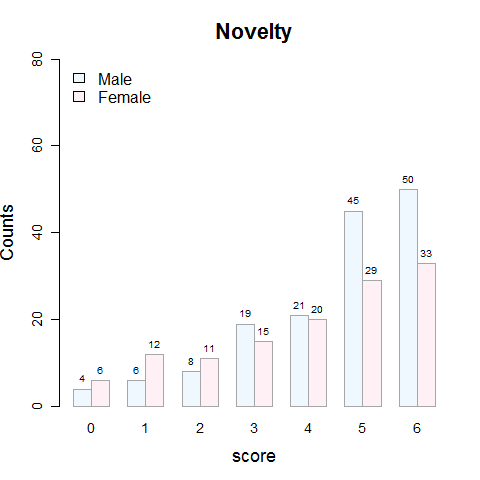

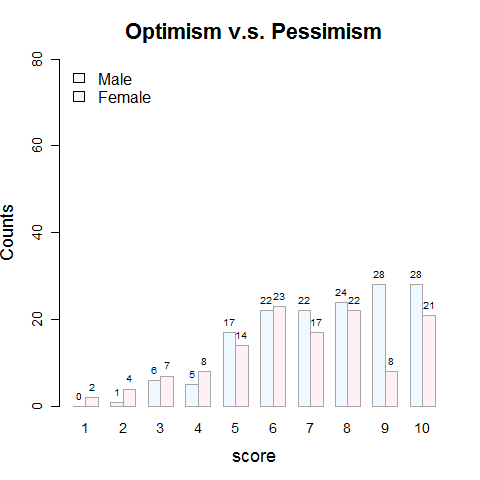

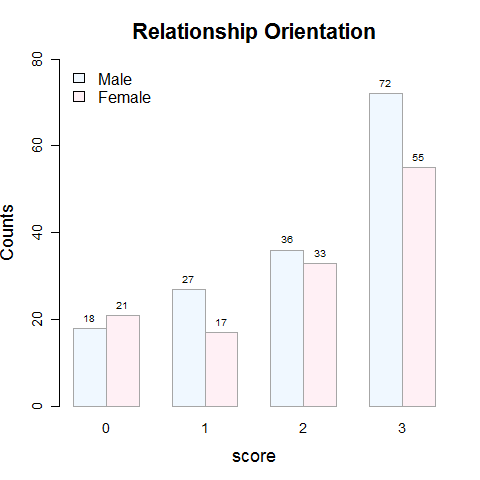

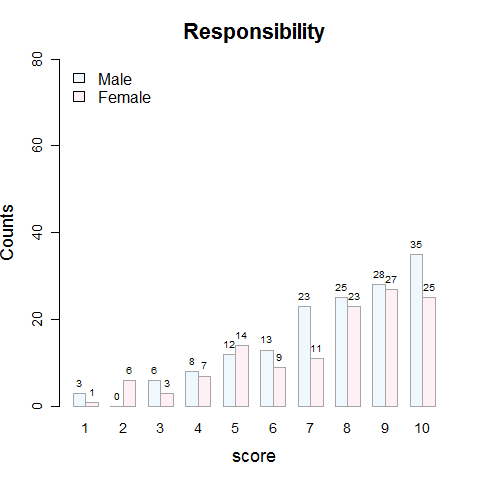

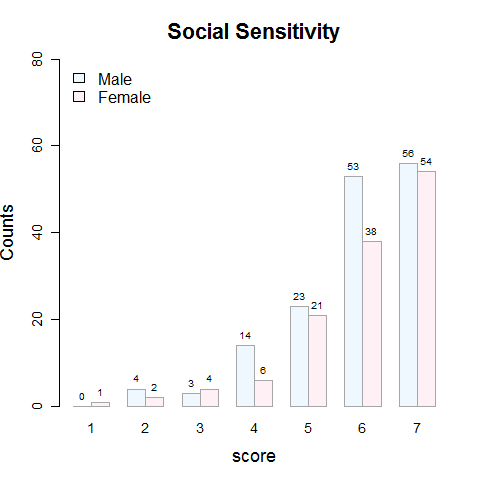

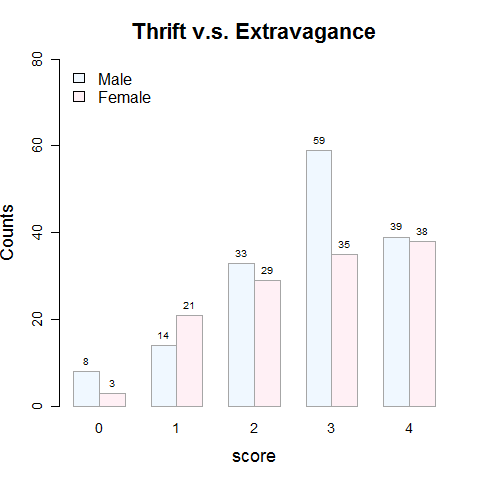

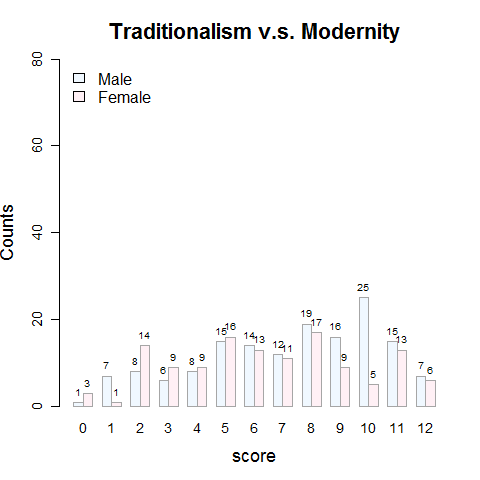


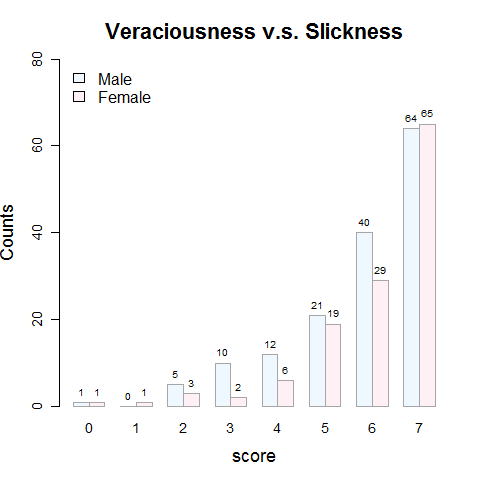


**Supplementary Fig S1. The distribution patterns of the 19 personality traits.**

Supplement: S1 Fig — (DOCX) [file pone.0281903.s001.docx]
