# Supplementary material for: A genome-wide association study (GWAS) of the personality constructs in CPAI-2 in Taiwanese Hakka populations
Source: PLoS One. 2023 Feb 17;18(2):e0281903. doi: 10.1371/journal.pone.0281903 (PMC9937499; doi:10.1371/journal.pone.0281903)
Supplement: S2 Fig — (DOCX) [file pone.0281903.s002.docx]

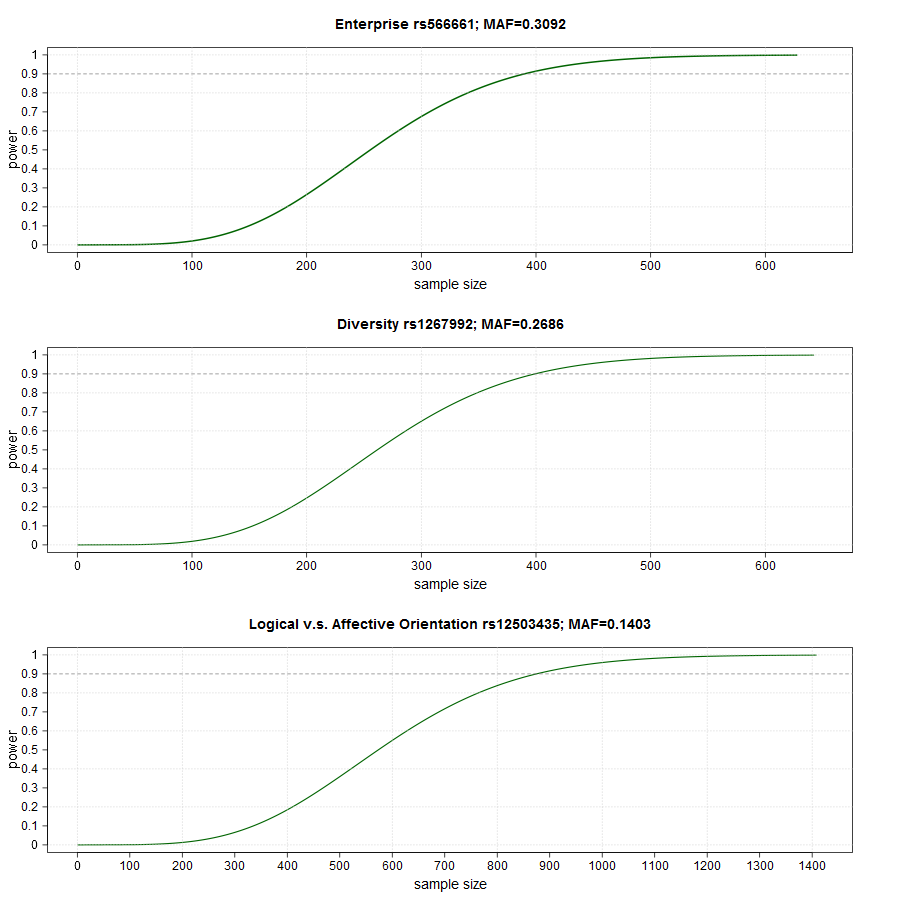


**Supplementary Fig S2. The plots of power by sample sizes for the three most significant SNPs shown in Fig 2.**
