# Supplementary material for: A genome-wide association study (GWAS) of the personality constructs in CPAI-2 in Taiwanese Hakka populations
Source: PLoS One. 2023 Feb 17;18(2):e0281903. doi: 10.1371/journal.pone.0281903 (PMC9937499; doi:10.1371/journal.pone.0281903)
Supplement: S3 Fig — The horizontal and vertical axes in each plot represent the theoretical and observed p-values in–log10 scale, respectively. (DOCX) [file pone.0281903.s003.docx]

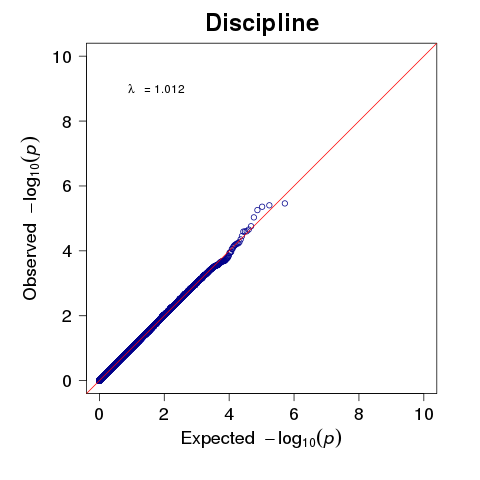

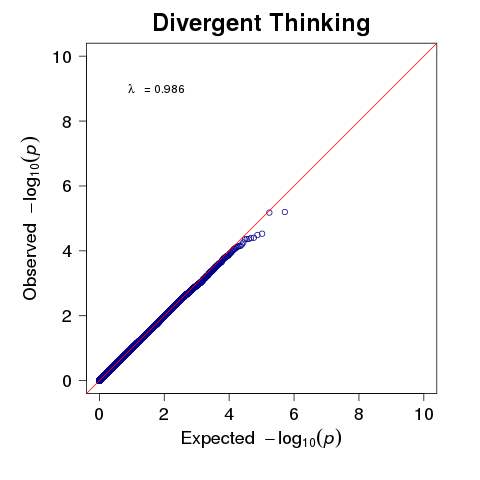

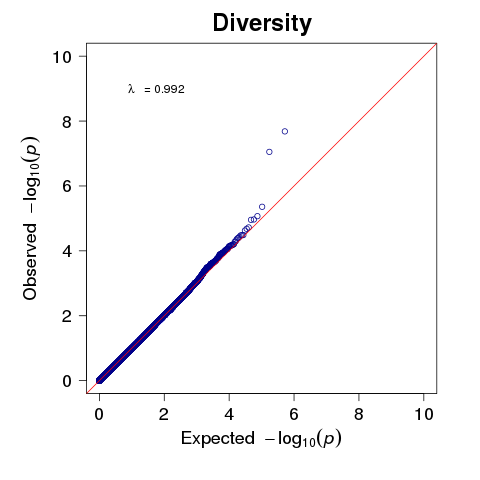


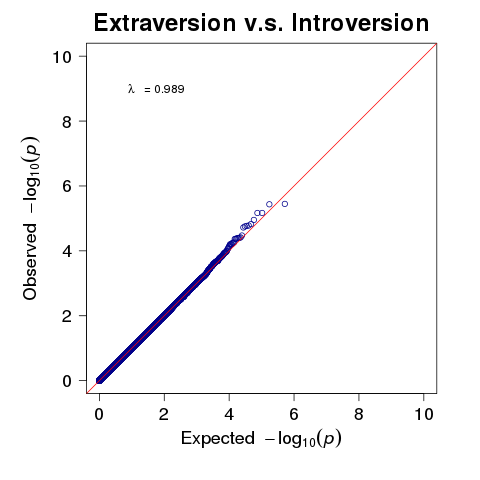

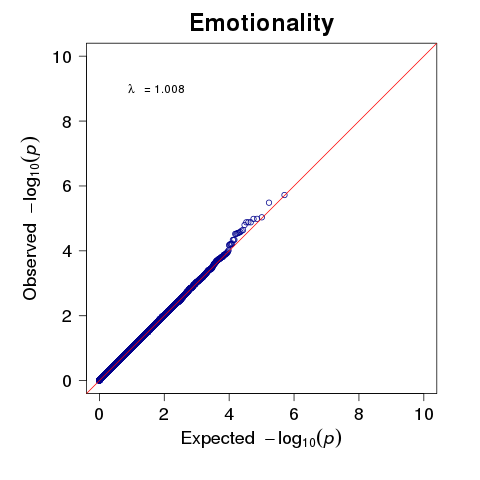

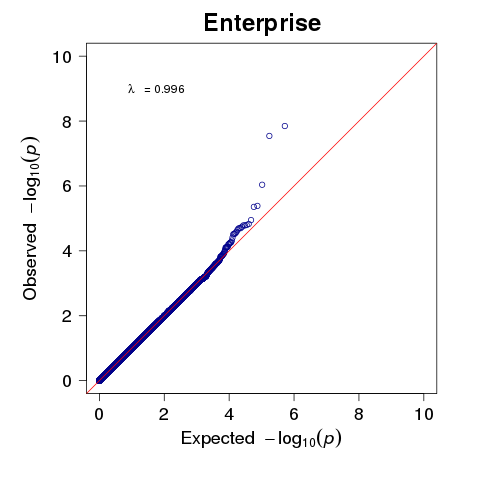


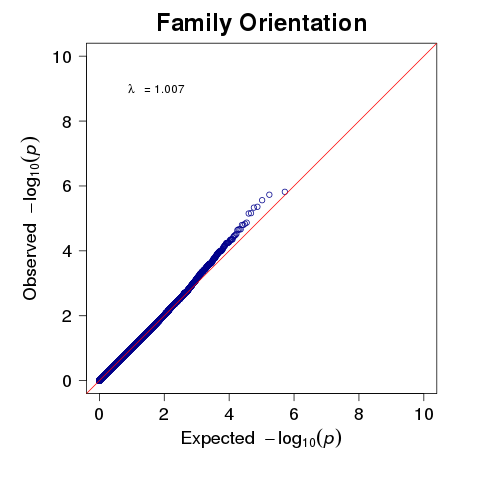

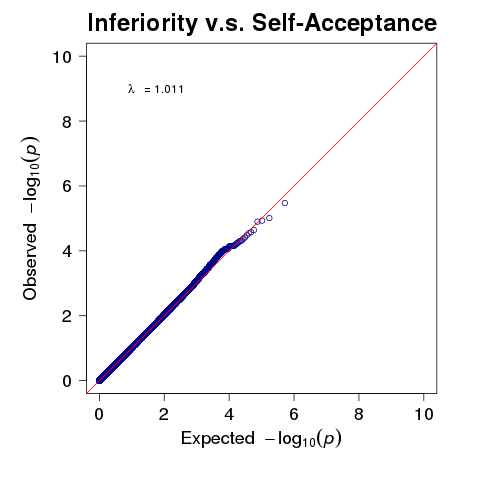

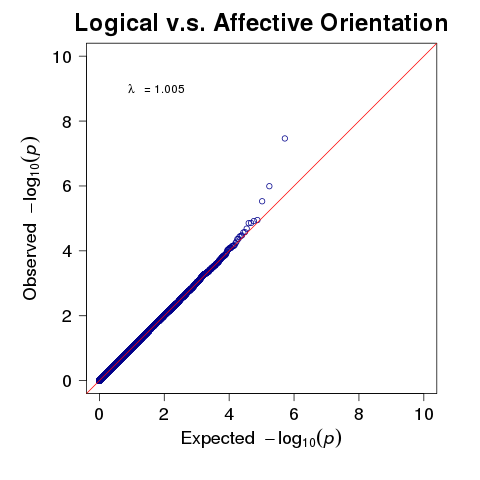


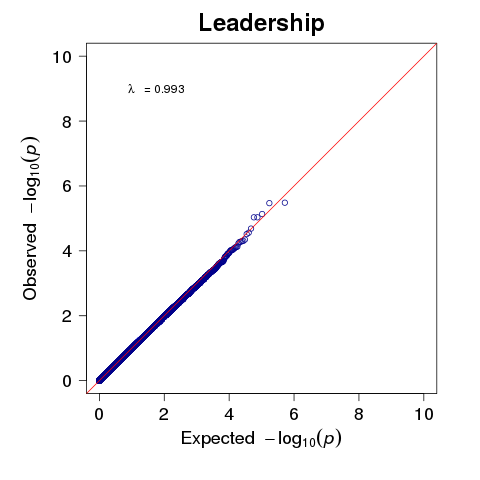

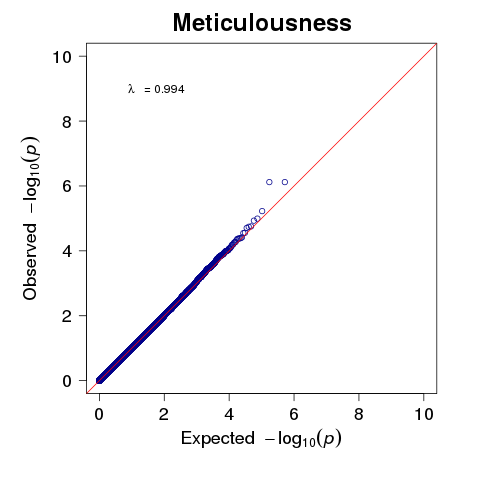

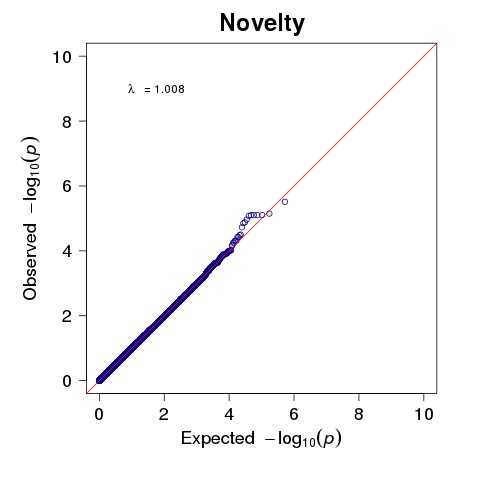


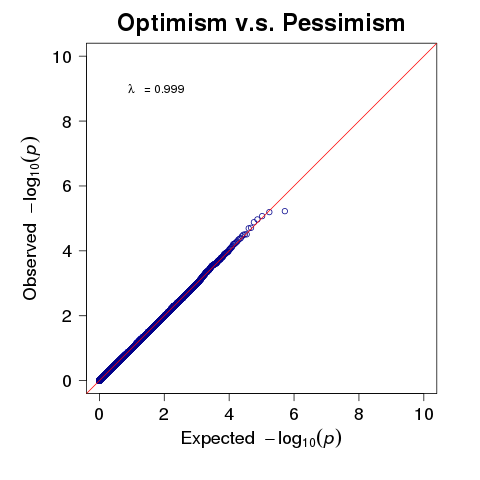

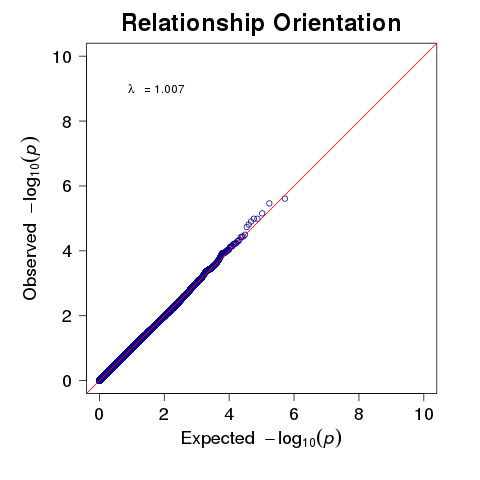

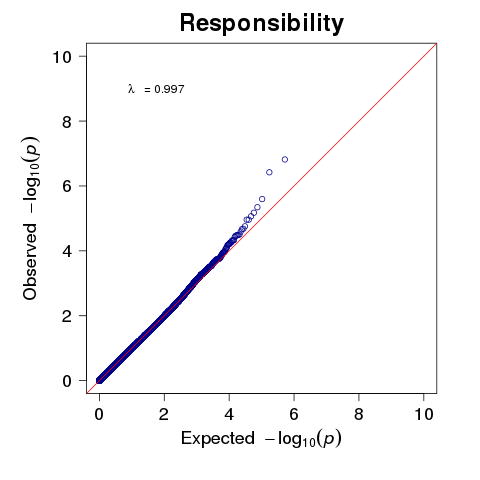


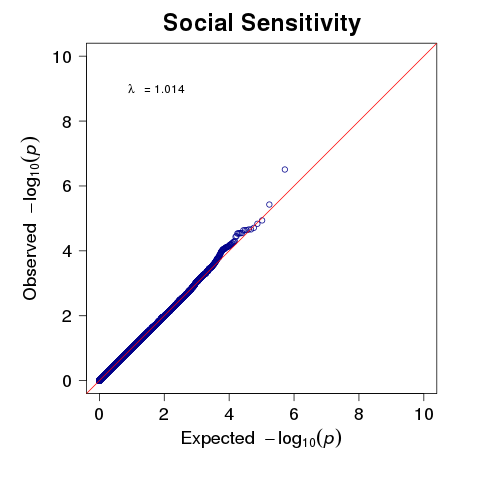

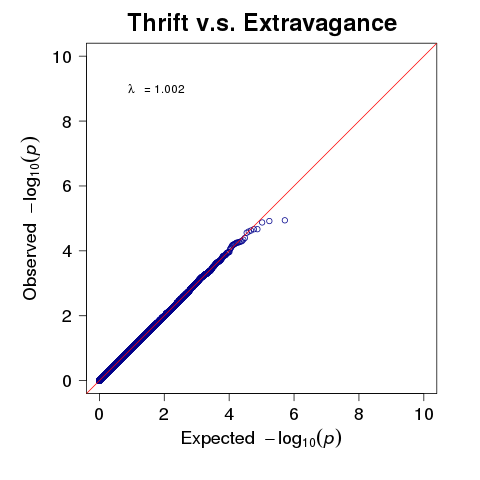

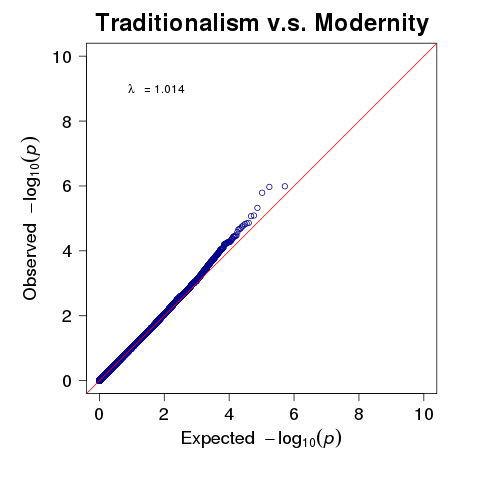

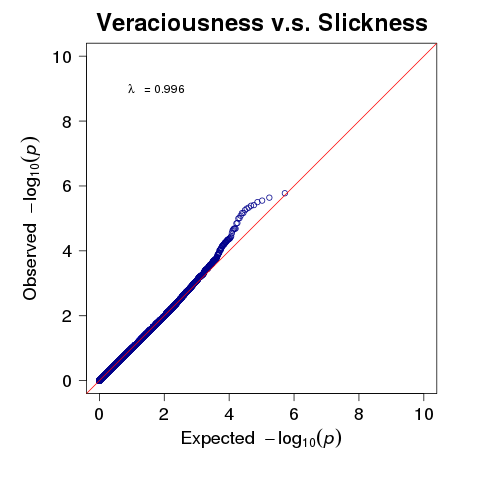


**Supplementary Fig S3. The quantile-quantile plots for the 19 personalities.**

The horizontal and vertical axes in each plot represent the theoretical and observed *p*-values in –log_10_ scale, respectively.
