# Supplementary material for: A genome-wide association study (GWAS) of the personality constructs in CPAI-2 in Taiwanese Hakka populations
Source: PLoS One. 2023 Feb 17;18(2):e0281903. doi: 10.1371/journal.pone.0281903 (PMC9937499; doi:10.1371/journal.pone.0281903)
Supplement: Attachment — Submitted filename: Response to reviewers.docx [file pone.0281903.s004.docx]

Reviewer #1: In this manuscript, the authors used GWAS to identify the associations between genetic variants with the personality constructs in the CPAI-2 among Taiwanese Hakka population. With 279 participants collected, the authors reported three loci associated with Enterprise, Diversity, and logical vs affective orientation, respectively. These loci are located with nervous-related genes nearby. It’s a very meaningful and reasonable investigation, and the manuscript is written very well. However, I still have some concerns with the GWAS analysis:

Major concerns:

1) Although the authors discussed about the potential low power of the study due to the small sample size and emphasized the findings after the type 1 error rate control, I think it’s still good if the authors could provide some figures or tables of power calculated with respect to different sample sizes and effect sizes (Based on the range of effect sizes you observed from collected data).

We used genpwr (Moore et al. 2019) to calculate the power by sample size for the three most significant SNPs. The results were provided in Supplementary Figure S2. The calculated power for the three identified SNPs are 0.597, 0.571, and 0.049, respectively. We have added a paragraph to provide this information:

We utilized the *R*^2^ values and genpwr [38] with linear and additive models to calculate the power for identifying the three most significant SNPs rs566661, rs1267992, and rs12503435 (Figure 3). The calculated values are 0.597, 0.571, and 0.049, respectively (Figure S2). These values are not quite high and imply that there might be many other personality-related variants remaining unidentified due to our limited statistical power.

The manuscript were revised accordingly.

Moore CM, Jacobson SA, Fingerlin TE. Power and sample size calculations for genetic association studies in the presence of genetic model misspecification. Human Heredity. 2019; 84(6):256-271. doi: 10.1159/000508558.

2) I can understand that it’s difficult for authors to collect another independent study for replication, could you also provide an estimated power/sample size for replication studies? So that the readers can get a sense how many individuals are needed to replicate the findings. You can refer the following paper for details:

Jiang, W. and Yu, W., 2016. Power estimation and sample size determination for replication studies of genome-wide association studies. BMC genomics, 17(1), pp.19-32.

Jiang and Yu (2016) estimated power and sample size using “case/control” models, which might be inapplicable for our linear regression analyses. We utilized the results mentioned in the above question to provide this information instead. We have added one sentence in the “Prospect” part:

Referring to the three SNPs identified in our study, the power to identify other SNPs with similar effect sizes could reach 0.9 as long as the sample sizes were enlarged to 400, 400, and 900, respectively (Figure S2).

3) In the discussion section, there is one paragraph describing why the commonly used genome-wide significance level (5*10^-8) is not adopted in the manuscript. The authors stated that the extent of the (multiple testing) problem is actually positively correlated with the number of examined SNPs. I can’t agree with this statement in the GWAS setting. Taking account of the LD structures and number of haplotype blocks across the human genome, we can estimate an effective number of tests, that is, the equivalent number of independent SNPs along the genome. The genome-wide significance threshold was proposed not based on the number of SNPs tested in a given study but rather on the number of SNPs in the whole genome that might have been tested. You can refer the following paper for details. So I would suggest the authors changing the statement of this paragraph and reporting results based on genome-wide significance level. The authors can still report the findings passing the Bonferroni correction as strong-evidence findings. In the Bonferroni correction, we should also consider the number of phenotypes considered, so the adjusted threshold should be 0.05/(19*260557)=1e-8 .

Jannot, A.S., Ehret, G. and Perneger, T., 2015. P< 5× 10− 8 has emerged as a standard of statistical significance for genome-wide association studies. Journal of Clinical Epidemiology, 68(4), pp.460-465.

Thanks for the kind reminder and suggestions. We have revised our manuscript accordingly.

4) For the 19 phenotypes, could you provide an estimation of genetic correlations and phenotypic correlations? And, what’s the heritability estimated from the collected data?

The Pearson correlation coefficients between each pair of the 19 phenotypes were listed below:

However, we did not find evidence for genetic correlations in our dataset. There are only 4 SNPs associated with two different personalities simultaneously with *p* value < 5 × 10^-5^

Among them, the correlation coefficient between EMO and FAM is only 0.153.

A larger sample size might be required to provide sufficient power to study the genetic correlation issues.

| SNP | chr | position | *p* value |  |
| --- | --- | --- | --- | --- |
| rs9310908 | 3 | 29745863 | 1.04E-05 | EMO |
| rs9310908 | 3 | 29745863 | 2.65E-05 | I_S |
|  |  |  |  |  |
| exm316983 | 3 | 50099500 | 1.32E-05 | EMO |
| exm316983 | 3 | 50099500 | 4.51E-05 | FAM |
|  |  |  |  |  |
| rs2830675 | 21 | 28460775 | 4.80E-05 | T_M |
| rs2830675 | 21 | 28460775 | 1.75E-05 | DIS |
|  |  |  |  |  |
| rs4965162 | 15 | 97804417 | 1.07E-06 | T_M |
| rs4965162 | 15 | 97804417 | 5.55E-06 | DIS |

The heritability contributed by single variants were estimated according to Zuk et al. (2012).

| Phenotype | SNP | Major allele frequency | Minor allele frequency | *h*^2^ |
| --- | --- | --- | --- | --- |
| Enterprise | rs566661 | 0.689964 | 0.310036 | 0.1128348 |
| Diversity | rs1267992 | 0.731183 | 0.268817 | 0.1054081 |
| Logical v.s. Affective Orientation | rs12503435 | 0.859712 | 0.140288 | 0.0495496 |

We have added a paragraph to provide this information:

The heritability contributed by these single variants could also be estimated from their allele frequencies and their effect sizes (defined as the regression coefficients) [56]. The estimated heritability values are 0.113, 0.105, and 0.050, which indeed supports the strong impacts of the identified variants on the analyzed Taiwanese Hakka populations.

Zuk O, Hechter E, Sunyaev SR, Lander ES. The mystery of missing heritability: Genetic interactions create phantom heritability. Proceedings of the National Academy of Sciences. 2012; 109(4):1193–1198. doi: 10.1073/pnas.1119675109.

5) If the phenotypes are genetically correlated, I would suggest the authors could consider conduct multi-trait analysis (e.g., MTAG) to improve power based on the summary statistics from each individual trait.

We performed MTAG multi-trait analysis (Turley et al. 2018) for the 19 personalities analyzed in our work. Both 1-vs-1 correlation and 1-vs-18 joint correlation scenarios were conducted. However, no significant results (*p* value < 5 × 10^-8^) were identified. The possible explanations might be: (1) MTAG was designed for meta-analysis and the power was largely derived from the combination of different datasets. In contrast, our 19 personalities were all obtained from the same dataset. (2) The 19 personalities were not essentially genetically correlated.

The 1-vs-18 joint correlation results were provided below:

Turley et al. "Multi-trait analysis of genome-wide association summary statistics using MTAG." Nature genetics 50.2 (2018): 229-237.

6) The SNP number after QC are almost half of the total collected SNP number. That’s unexpected. Could you also provide the filtered SNP number in each QC step? So that we can get a sense of the majority of SNPs were filtered due to which reason.

Step 1: Four types of SNPs were filtered out utilizing R programming:

(1) insertion / deletion polymorphisms (12,524 SNPs removed)

(2-1) genotyped variants with the Chr. marker labeled as “0” (803 SNPs removed)

(2-2) genotyped variants with the Chr. marker labeled as “X” (12,943 SNPs removed)

(2-3) genotyped variants with the Chr. marker labeled as “Y” (1,934 SNPs removed)

(2-4) genotyped variants with the Chr. marker labeled as “XY” (208 SNPs removed)

(2-5) genotyped variants with the Chr. marker labeled as “MT” (369 SNPs removed)

(3) & (4) missing rate > 5% and positional duplicates (a total of 11,364 SNPs removed)

A total of 507,499 SNPs were retained after this first step.

Step 2: Two other types of SNPs were filtered out utilizing PLINK:

(5) HWE *p*-value < 1 × 10^-5^ (74 SNPs removed)

(6) MAF < 1% (246,868 SNPs removed)

A total of 260,557 SNPs were retained after this second step.

The manuscript was revised accordingly to provide this information.

7) For association analysis, did you incorporate gender and PC (principal components) as covariates into the regression? If not, why not?

According to Cheung et al. (2004), the gender factor is supposed to have no influence on the designed personality traits. In our dataset, we also found that male and female have similar distribution patterns of the 19 personality traits (Figure S1, also shown in the response to Reviewer 2). On the other hand, the genotypes of the SNPs should not be associated with the gender. Therefore we did not incorporate gender as covariates into the regression.

As for the principal components, we assume that the principal components referred by the reviewer were transformed from the 19 personality traits. However, the aim of this study is to investigate the association between each personality trait and the genotypes. The transformed PC contains all the 19 personality traits and it would be difficult to interpret the results with PC as the covariates. On the other hand, as described above, we did not find evidence for genetic correlations, and no significant results were identified from the MTAG analysis. Therefore we did not incorporate PC as covariates into the regression either.

Cheung, F. M., Cheung, S.-f., & Jianxin, Z. (2004). What is "Chinese" personality? Subgroup differences in the Chinese Personality Assessment Inventory (CPAI-2). Acta Psychologica Sinica, 36(4), 491–499.

Reviewer #2: Distribution of Chinese personality Assessment Inventory 2 (CPA-2) of 288 recruited participants (159 males and 129 females) was not shown as histogram and the normality of the data we could not see for CPA-2. Authors should provide distribution pattern of CPA-2.

The distribution patterns of the 19 personality traits were provided in Supplementary Figure S1 and the manuscript was revised accordingly. Although the CPAI-2 personality scale has shown good reliability and validity among the Chinese population in Hong Kong and Mainland China, it has not been formally validated in Taiwanese population. In the current study, we conducted the confirmatory factor analysis to examine the validity of the CPAI-2, and therefore some items were deleted based on the statistical criteria. In this case, though we cannot compare the revised CPAI-2 with the original one, the revised CPAI-2 keeps an appropriate validity.
